# Of the Veterinary Pharmacopæia

**Published:** 1818

**Authors:** James Carver

**Affiliations:** Professor of Veterinary Medicine, and Corresponding Member of the Veterinary Medical Society of London and British India


OF THE

### VETERINARY PHARMACOPÆIA,

OR

### INSTRUCTIONS

FOR

### *Compounding Horse Medicines;*

AND

*Preparing the Various Substances employed in Veterinary Practice at the different Veterinary Colleges of*

### EUROPE AND BRITISH INDIA:

WITH

A LARGE COLLECTION OF VALUABLE RECEIPTS, OF ESTABLISHED EFFICACY, TRIED ON CONDEMNED ANIMALS AT THOSE INSTITUTIONS.

BY JAMES CARVER,

Professor of Veterinary Medicine, and Corresponding Member of the Veterinary Medical Society of London and British India.

### On Horse Balls.

Horse Balls are the most common, and generally the most convenient form in which medicines are given to horses. In giving a ball, where the balling iron (an instrument used by the common farrier) is not used, the tongue should be drawn out first by the right hand, and then laid hold of, over-handed, by the left, and so drawn over the grinders, on the left, or off-side. The giving of a ball is a forcible operation; and when necessary for a ball to be repeated very often, it may be dissolved in some gruel, and given as a drink. The most convenient mode of giving a ball is, to back the horse in a stall, and do as directed above: laying hold of the ball in the fingers of the right hand, which must be squeezed into as small a space as possible; the ball must be passed up the roof of the mouth, and left on the root of the tongue. The hand may then be withdrawn, and the tongue liberated, which will have the effect of throwing the ball further back on the œsophagus, or valve of the epiglotis, or gullet, when the ball will pass down. The head should be but moderately elevated, or much mischief may occur, in choking the horse. In giving the ball—which, to most horses, may readily be done by one person, but by two it is more convenient—the helper, who should stand on the near, or left side of the horse, should put his thumb between the tusk and grinder of the upper jaw: he should then take his two fore-fingers of his right hand, and place them between the tusk and grinder of the lower jaw; and by pressing the left hand up, and right one down, with the assistance of the operator on the other side, the mouth is considerably and widely opened, quite sufficient to admit of the *right hand and arm* of the person who gives the ball: when the operator has delivered the ball, and at the same time withdrawn his hand and arm, the signal *let go* is given; and the person who assists lets go at the same time that the operator does the tongue. The ball, if properly placed, is then swallowed by the horse.*

A horse ball should never be too large, nor too hard. The size of a pullet's egg, only rather longer, is quite sufficient: they should never be larger.

Before I left the Veterinary College, Mr. Long, of High Holborn, London, invented a very ingenious instrument for giving balls, which, for the small class of horses, such as poneys, galloways, &c. is very convenient for horses with a small mouth. They are rather an expensive instrument; and it may be a long time before they come into use in this country. A person desirous of learning how to deliver a ball, may learn it in a few hours, if so disposed; by making up a few balls, of common bread dough, and practising on a few horses with wide and open gullets. This method will soon give him confidence: a few trials will, of course, make him expert.

There are some circumstances in the preparation of horse medicines not sufficiently attended to. Balls, by keeping, become hard, and are liable to stick in the gullet, and endanger life; and when composed of camphor, ammonia, or any of the essential oils, their virtues evaporate: they should, therefore, be made as wanted.

When a ball is composed of a very stimulating ingredient, such as corrosive sublimate, arsenic, and many others too numerous to enumerate, a little drink, such as gruel, &c. should be prepared, to prevent a too strong and hasty action on the stomach. All kinds of oils should be given with the greatest caution and patience, and well mixed with good palatable gruel, to prevent its disagreeable effects on the taste of the animal; for I know of no medicine that is administered to a

* If the horse should cough the ball up, it must be again rolled up in a fresh piece of paper and re-administered.

horse that he takes with more reluctance than oil, particularly if bitter or rancid. I have, during my practice, known horses after a quart of oil has been given them, to have been so disgusted with its taste, as not to permit a man to come near them for a week. The best mode of preparing and keeping for use horse medicines, is to have the mixture made, and put into a jar covered over, for use, with a bladder.

---

### Barbadoes Aloes.

(See ALOES.)

---

### Balsams.

Of the balsams used in Veterinary medicine, there are a variety which are more or less used under the following heads, viz.

| | |
|---|---|
| Balsam of *Sulphur*. | Balsam of *Capeva*. |
| Balsam of *Gilead*. | Balsam of *Tolu*. |
| Canada *Balsam*. | Balsam of *Peru*. |

Balsams are generally fluid, and of various degrees of thickness, united with the extractive matter of the various plants they are obtained from: they are occasionally used in Veterinary medicines. The balsam of Canada is a very pure kind of turpentine, though rather expensive for Veterinary practice. It is, however, an excellent diuretic in the dose of one ounce or more. The Venus turpentine is, I believe, equally efficacious, and much cheaper. The balsam of sulphur has been most in use among farmers and farriers, and considered by them as an expectorant very highly improper, though useful in chronic cough.

### Barrilla,

Is a sea plant; from the ashes of which the mineral alkali, or soda, is obtained in an impure state.

---

### Barbadoes Tar,

Is a bituminous tar substance, brought from the island of Barbadoes: It is very nearly the same colour and consistence of common tar; but its colour approaches more to a brown, and possesses a diuretic quality. Farriers use it indiscriminately, and thereby do much mischief. At some of the colleges it is more highly valued as an internal medicine for coughs. In obstinate chronic coughs, I have experienced its good effects, when joined with expectorants, and alteratives, particularly of the mercurial kind.

---

### Barks,

In the human subject, is used as a tonic; and though by some Veterinarians not thought to possess the same effects on the horse, I have uniformly administered it under various forms, with very good effect. In fine, all the barks which constitute the Veterinarian's list of medicaments, act with an astringent property. In fine, the barks of this country are, for many veterinary purposes, invaluable; and I believe I should not overrate their virtues to say that many of them are considerably more valuable than the Peruvian, or any of the barks used in common on the human subject. For instance, the *oak*, the *elm*, the *quassia*, the *sumach*, the *persimmon*, and many others too numerous to mention, the common growth of our own woods, which I consider as invaluable to the practice of the Veterinary Surgeon and Physician; possessing all the tonic qualities internally administered, as well as the astringent qualities, when externally applied, and not near so expensive. I have both seen and experienced the wonderful effects of many of these barks, both externally as well as internally applied, in *gangrene*, in diabetes—*diarrhœa:* and when mixed with *opium*, *ginger*, *ammonia*, and other medicines known to the Veterinarian, may be very usefully applied. In cases of debility, where the discharge of matter is copious, arising from large suppurations, I have used it very successfully. The dose is from one to six ounces, according to the different strengths and tannin qualities or principles of the bark used.

All these barks may be usefully applied in diseases among horned cattle.

---

### Basilicon.

(CERATUM RESINEÆ.)

The yellow basilicon, the white basilicon, the warm stimulating basilicon, the black basilicon, and many others, are used in Veterinary practice, for dressing wounds.

---

### Benzoin:

Or what is commonly called Gun Benjamin, is used in Veterinary medicine as a compound tincture in friars balsam, or " in tincture" with aloes.

---

### Blisters,

Are of the greatest importance in Veterinary medicine; nor could we succeed in performing many of our cures without them, though there is both judgment and knowledge required in their use and application.

When blisters are properly made, and free from the caustic ingredients, such as sublimate, and many of the acids used by the common farrier, grooms, &c. there is no danger of destroying the hair; which gives the Veterinarian, who, having a proper knowledge of his ingredient in making of blisters, the power of a frequent application of them when necessary. Blistering is considered the best application for *curbs*, *spavins*, *wind-galls*, and various internal inflammations; and when properly applied in this way, act by a counter irritation, and thereby, particularly on inflamed lungs, very often saves the patient. The substances most used for this purpose are the Spanish flies, which, when properly mixed with other ingredients, makes their action strong or mild, according to the action or disease they may be wanted for.

EUPHORBIUM—a substance sometimes introduced as a substitute for Spanish flies; but, by causing too much irritation, should never be used.

---

*Blisters for general use.*

| | |
|---|---|
| Take of Pulv. cantharides . . . | 6 oz. |
| Venice turpentine . . | 3 |
| Spts. do. do. . . . | 3 |
| Resin . . . | 4 |
| Lard or Palm oil . . . | 1 lb. |

| | |
|---|---|
| Take of Cantharides . . . | 1 lb. |
| Oil or lard . . . | 2 |
| Oil of turpentine . . . | 6 oz. |
| Oil of vitriol . . . | 2 dr. |
| Pul. euphorbium . . . | 3 oz. |

Mix for a cheap blister.

### *Strong Blister.*

(For Chronic Swellings, or Diseases of the Bones.)

| | | |
|---|---|---|
| Take of | Pul. Spanish flies | 1 oz. |
| | Oil of turpentine | 1-2 |
| | Venice do | 1-2 |
| Mix | Resin and lard, each | 2 |

| | | |
|---|---|---|
| Take of | Oil of Tereb. | 1 oz. |
| | Vitriolic acid | 2 dr. |
| | Lard | 4 oz. |
| | Spanish flies | 1 |

Mix carefully in an open chimney.

---

### *Stronger Blister.*

| | | |
|---|---|---|
| Take of | Mercurial oint. | 4 oz. |
| | Spanish flies | 1 1-2 |
| | Venice turpentine | 4 |

Lard, quant. suff. to mix.

| | | |
|---|---|---|
| Take of | Spanish flies | 1 oz. |
| | Barbadoes tar | 1 |
| | Oil of thyme | 2 dr. |
| | Mercurial oint. | 2 oz. |

---

### *Mild Blister.*

| | | |
|---|---|---|
| Take of | Lard | 1 lb. |
| | Venice turpentine | 6 oz. |
| | Spanish flies | 6 dr. |

Mix.

| | | |
|---|---|---|
| Take of | Cantharides | 1 oz. |
| | Mercurial oint. | 1 dr. |
| | Venice turpentine | 4 dr. |
| | Lard | 4 oz. |

Mix.

### *A Liquid or Sweating Blister.*

| | | |
|---|---|---|
| Take of | Spanish flies . . . | 1 lb. |
| | Oil of turpentine . . . | 3 qts. |
| | Olive oil . . . . | 1 |
| Take of | Cantharides . . . | 1-2 lb. |
| | Oil of turpentine . . . | 5 pts. |
| | Olive oil . . . | 3 |

Mix.

*Note.*—For the strong blister, when oil, and resin, and vitriol is used, you must first melt the resin and the oil, and afterwards add the turpentine, having previously mixed the vitriol, gradually, with an oz. of water; which must be slowly added to the mass, by a very slow fire for ten minutes; let it cool, and then add the powders. For the liquid blisters, you must steep the flies in the turpentine for two or three weeks; then strain and add the oil.

---

### *A Mild Liquid Blister.*

| | | |
|---|---|---|
| Take of | Spanish flies . . . | 6 dr. |
| | Oil of turpentine . . . | 3 |
| | Olive oil . . . | 1 pt. |

Mix.

| | | |
|---|---|---|
| Take of | Spanish flies . . . | 3 dr. |
| | Mustard . . . | 1 oz. |
| | Spirits of turpentine . . | 3 dr. |
| | Olive oil . . | 1 1-2 pt. |

---

### *Mustard Blisters.*

| | | |
|---|---|---|
| Mix | Mustard . . . . | 8 oz. |
| | Water . . . | quant. suff. to make a paste |
| | Oil of turpentine . . , | 3 oz. |
| Mix | Mustard . , . . | 1 lb. |
| | Oil of turpentine . . . | 2 1-2 oz. |

Mix the latter with the mustard, after being made into a paste.

*Note.*—To either of the former you may add of pure ammonia one ounce; to be mixed and rubbed into the part wanted. If the bowels be affected, as in inflammation of the bowels or intestines, rub it all over the belly with the hand. If the kidnies over the loins, the friction should be continued for some time. The more it is rubbed, the more and sooner will it take effect; sometimes to a considerable degree, which acts by counter-irritation, and greatly diminishes the internal inflammation, and saves the animal's life. These are a most important class of remedies, and are the most effectual applications for removing those swellings and lameness, which a.e sometimes the consequence of bruises, strains, &c. they should not, however, be applied for lameness, or bruises, or open joint, when the inflammation is very great.

When, however, blisters are used for internal inflammation, this precaution is not necessary.

---

### Horse Beans, Split Peas, &c.

Are used as an article of diet, and are, perhaps, when judiciously mixed with other provender, one of the strongest and best foods we have in use. They are strongly astringent, and are excellent, both for horses and horned cattle in cases of excessive purging.

---

### Blue Vitriol, Blue Stone;

OR,

### (VITRIOLATED COPPER) OR CUPRI SULPHAS.

The sulphate of copper is, in Veterinary practice, a valuable tonic. I have used it at the Veterinary college with very beneficial effects, both internally and externally. It is also extremely useful, applied as a mild caustic, and is an excellent application for ulcers of every kind, disposing them to heal, sooner than any other application. The best way of using this article in solution is to pulverize it, and put as much of the powder in a pint of water, as it will dissolve, and boiling water will facilitate its solution. This solution may be used, mixed in this way, or diluted, as circumstances may require. When used in the powder, it is sprinkled on the sore. Blue Vitriol, as an internal remedy, certainly possesses a tonic power—but it requires caution in its administration. When administered internally it should be begun with small doses of half a drachm in the form of a ball. In cases of broken knees, I have used it at the college, with great success, particularly when applied hot.

---

### Bole Armenian,

Is an argillaceous earth, or red clay, impregnated with iron, and used by the common farrier, as an astringent in diarrhœa, bloody urine, &c.; formerly its qualities were much extolled as a strengthener, both externally and internally applied. It, however, now claims but little attention, except as a colouring matter.

